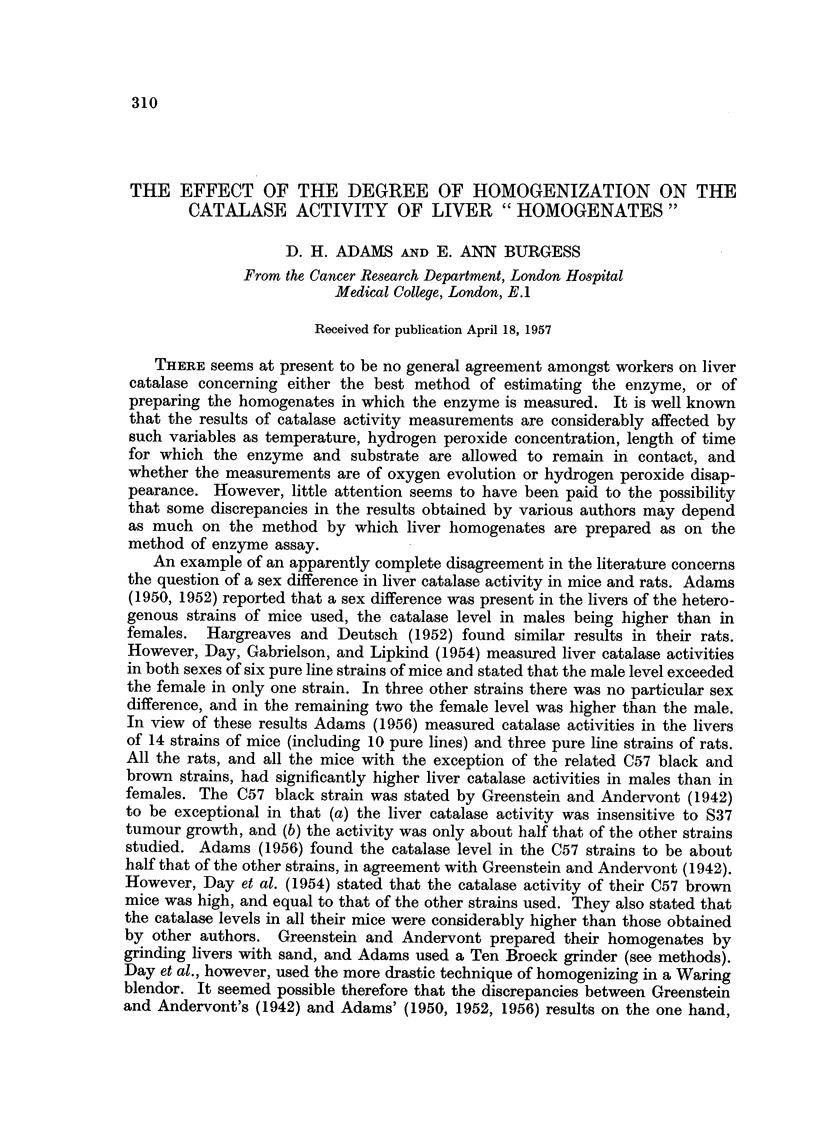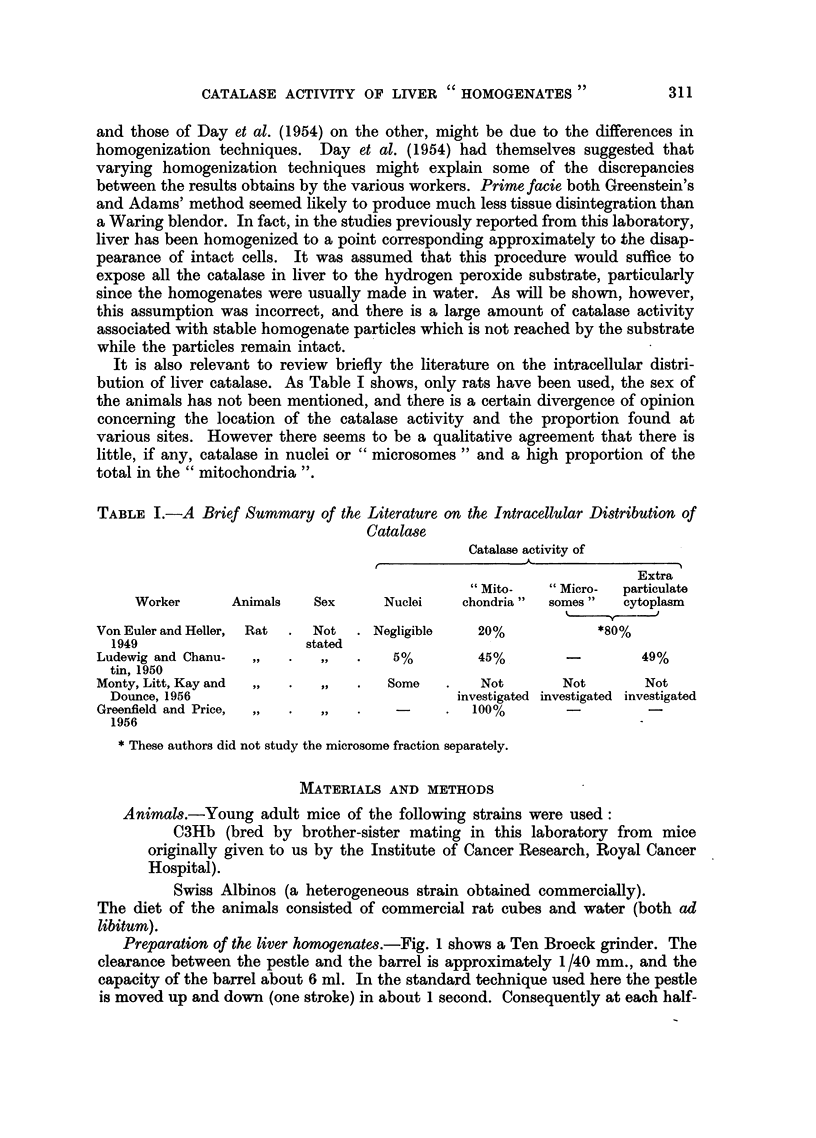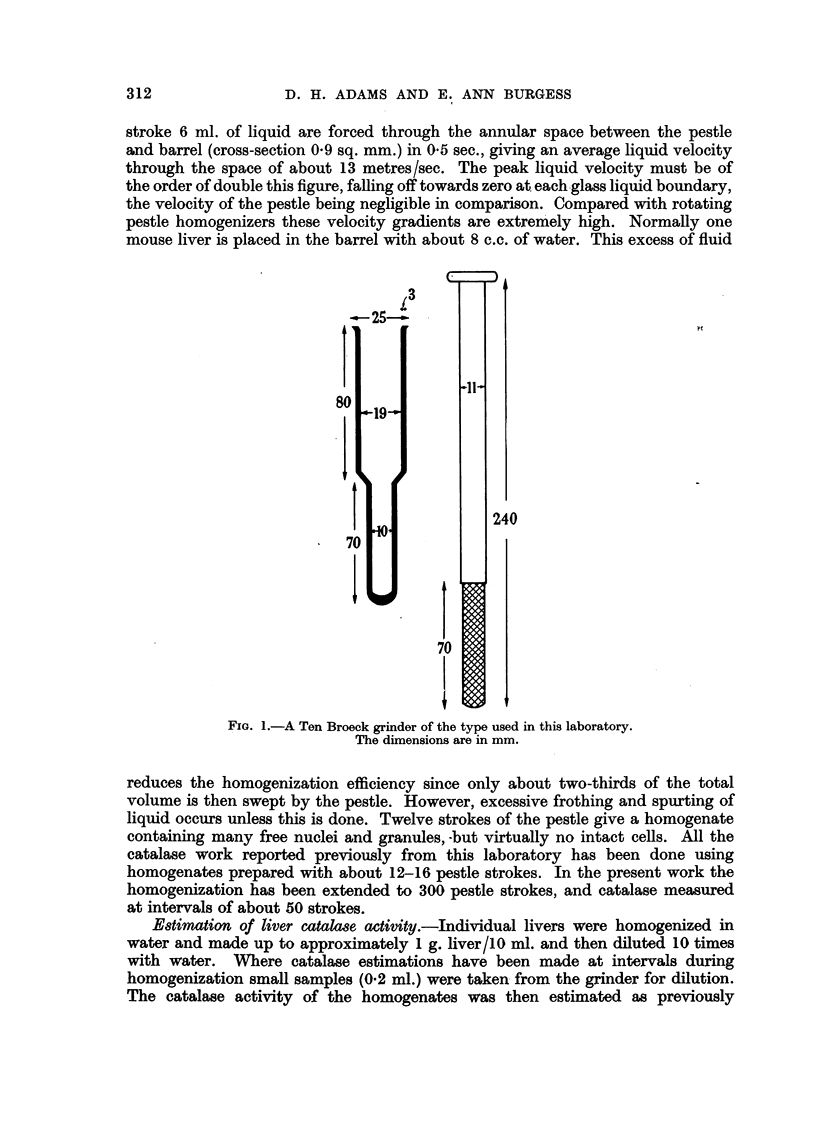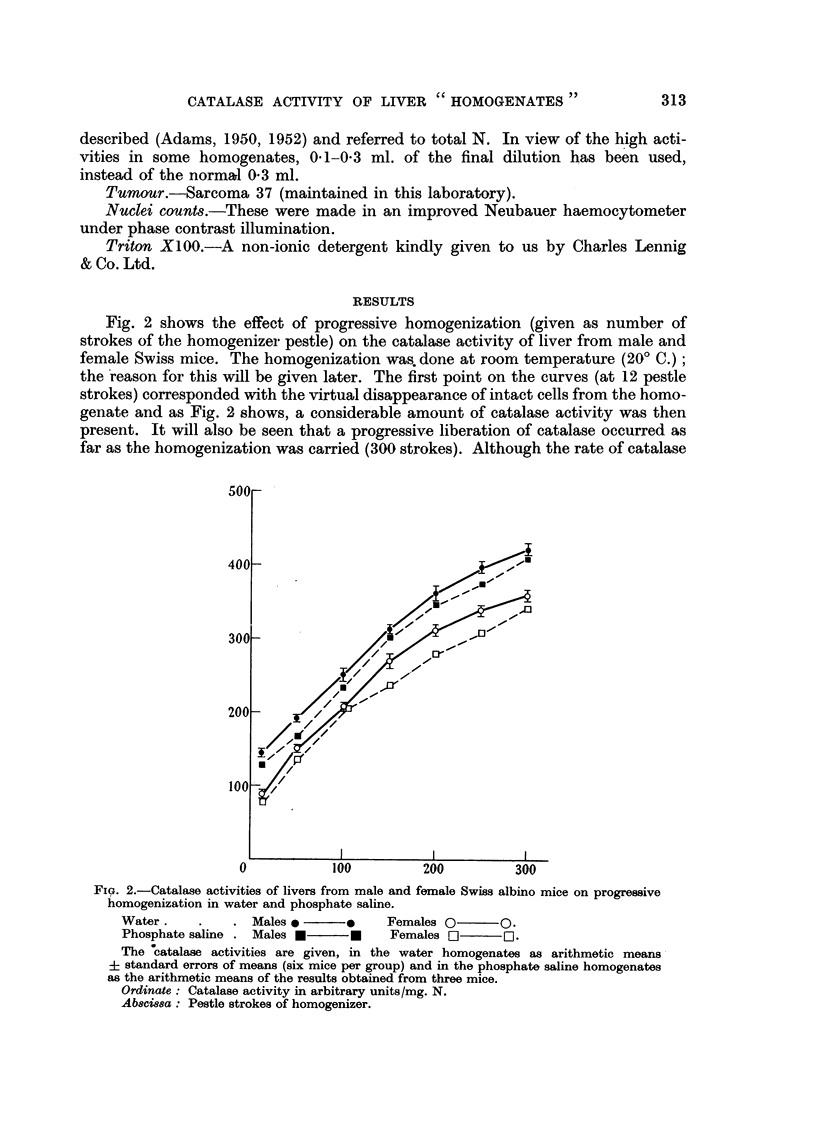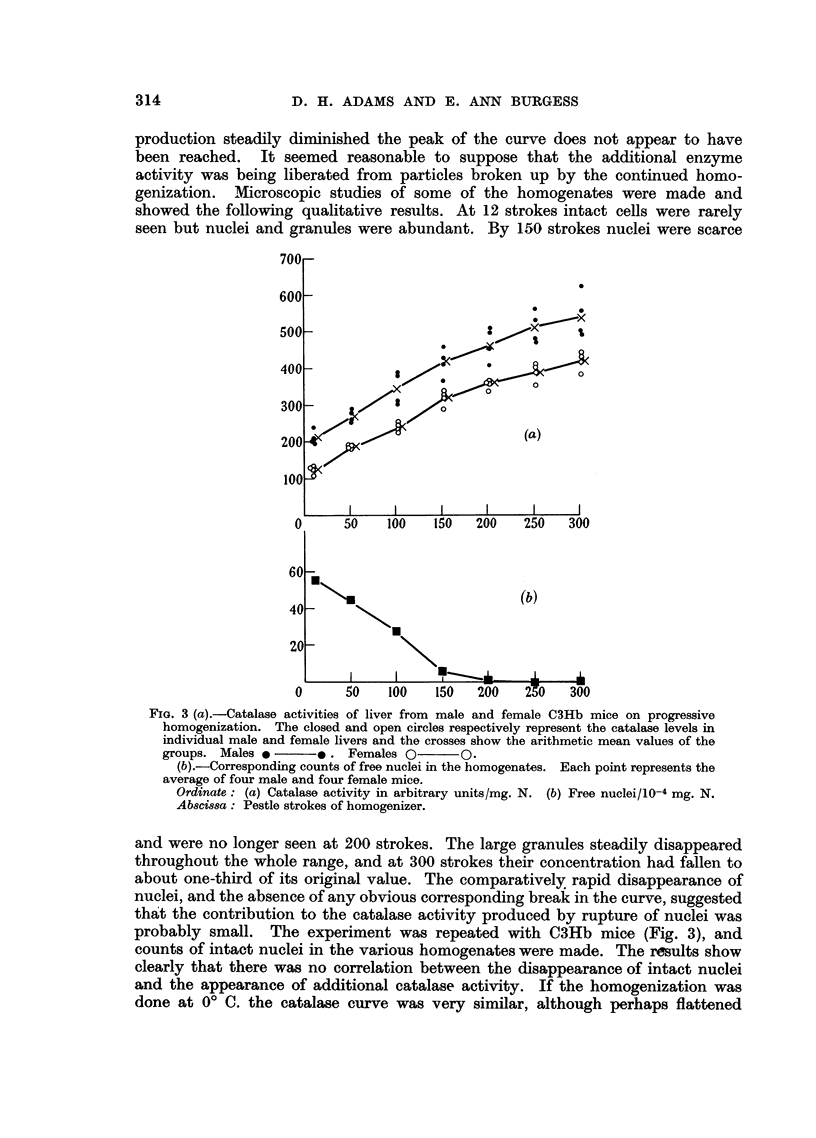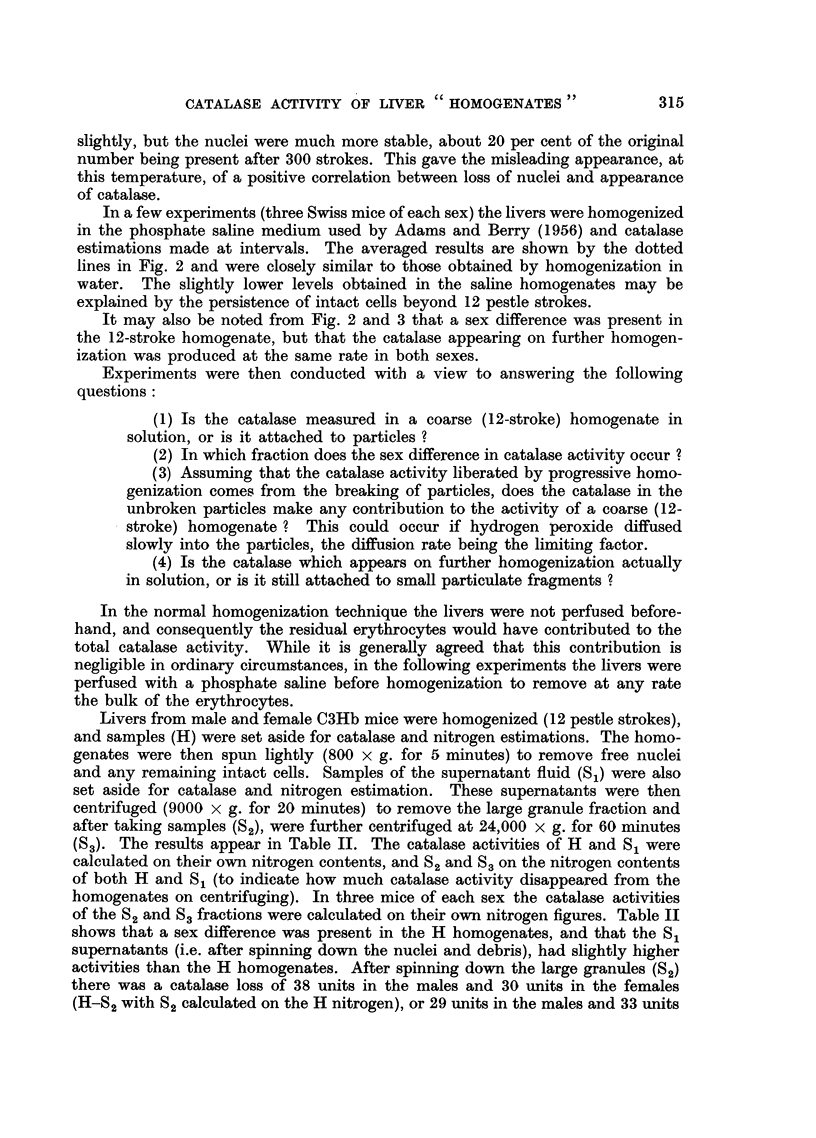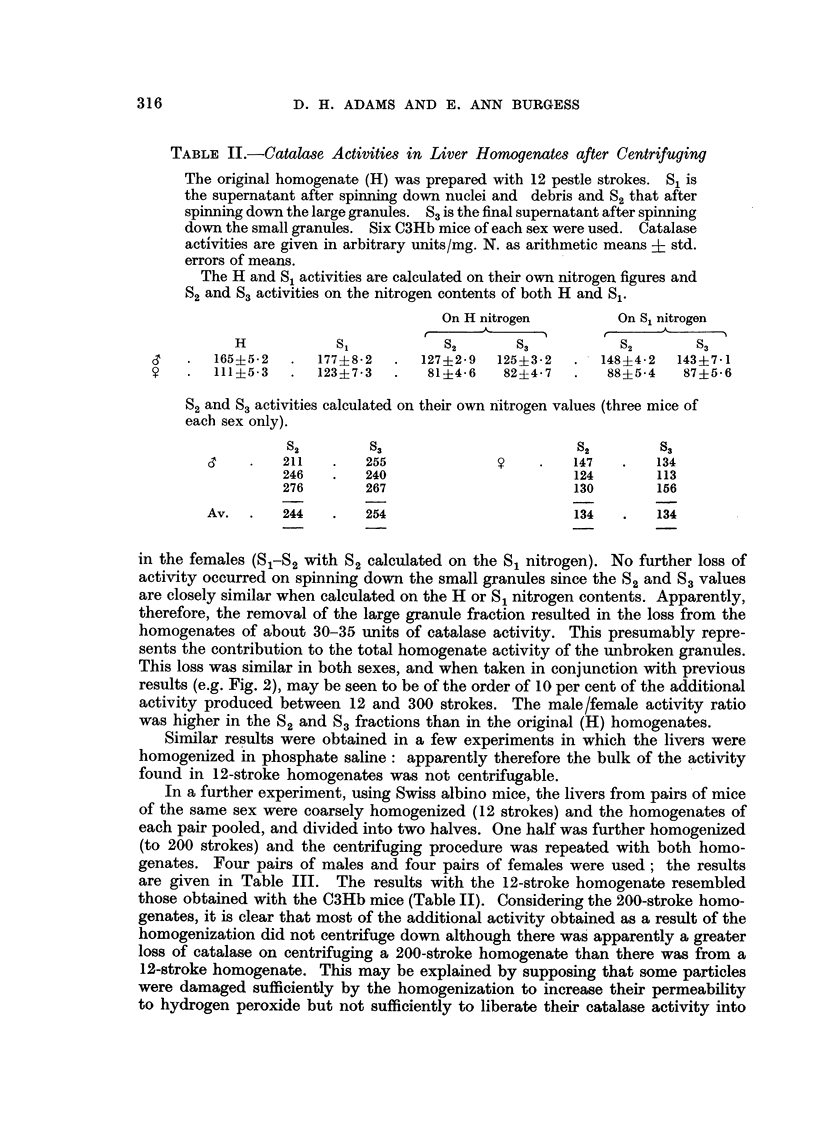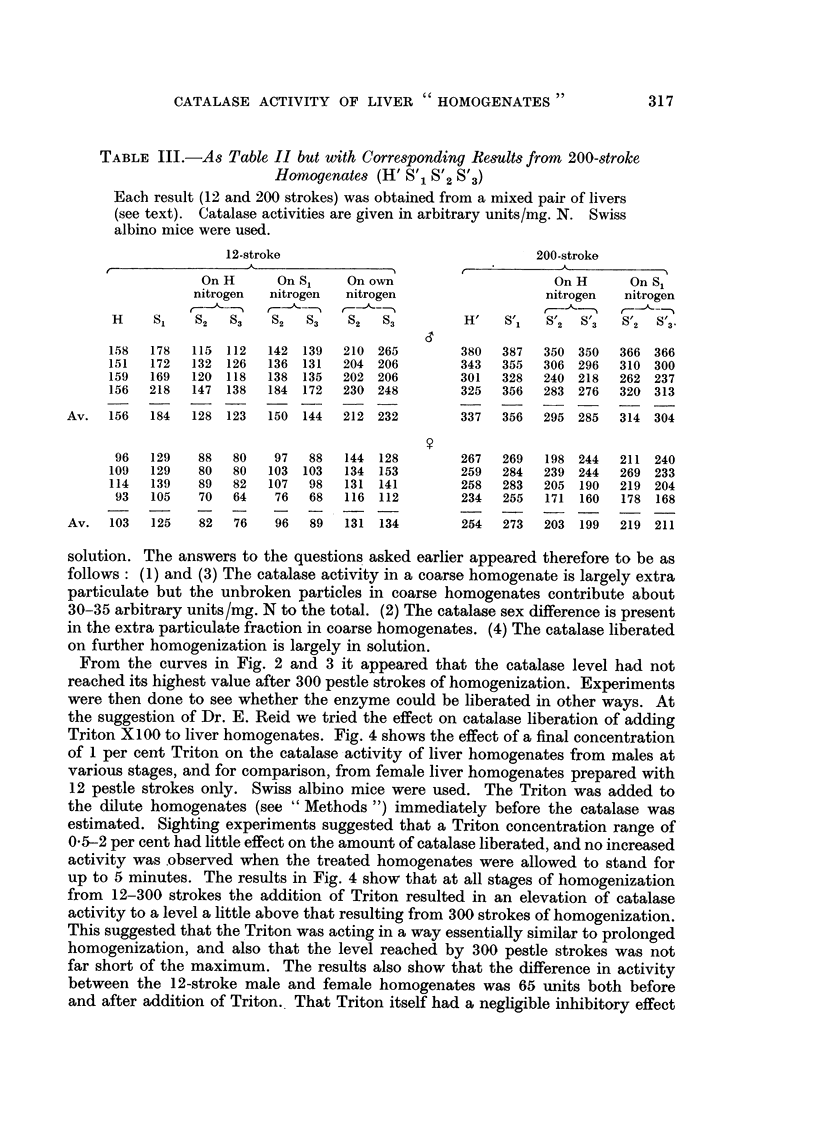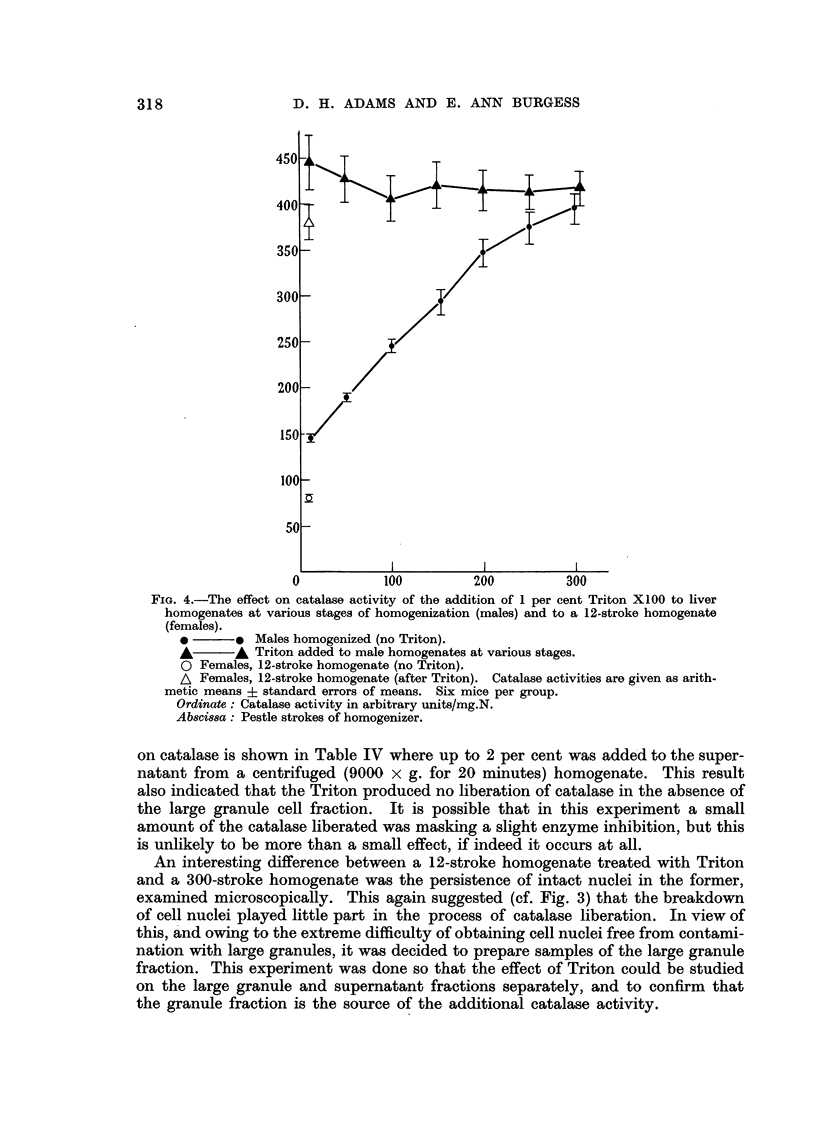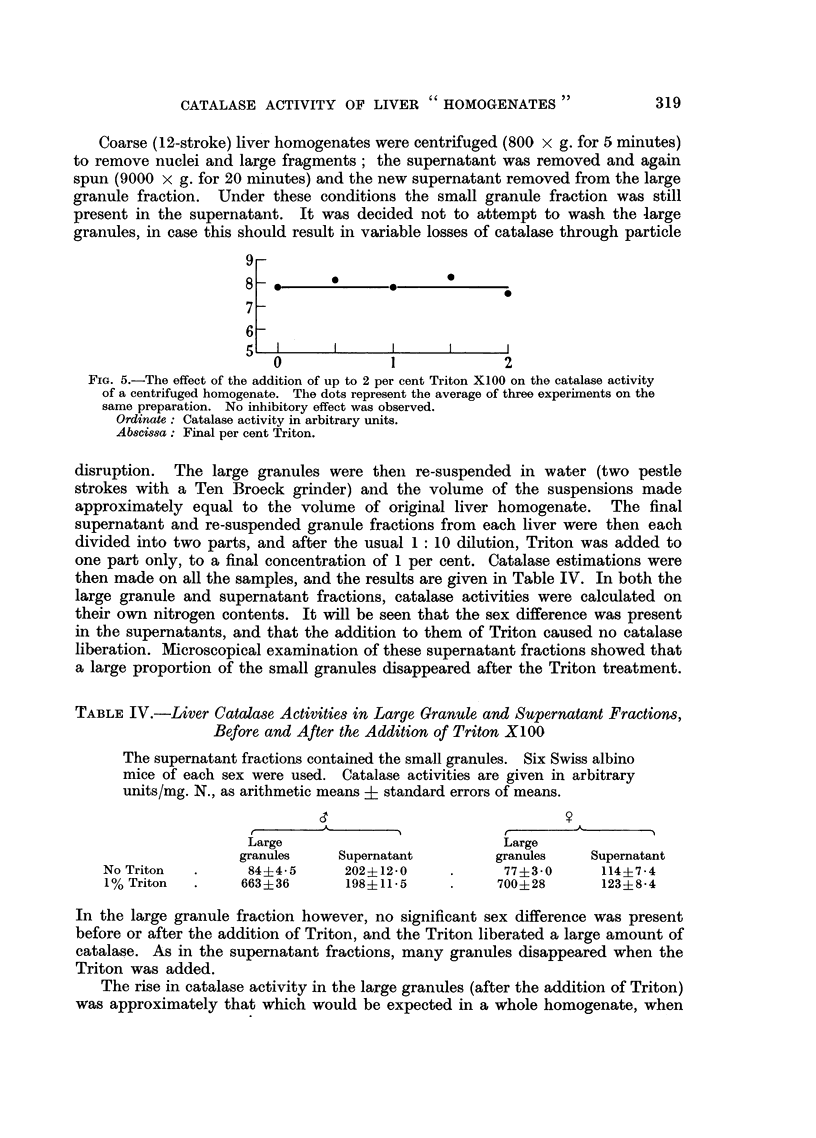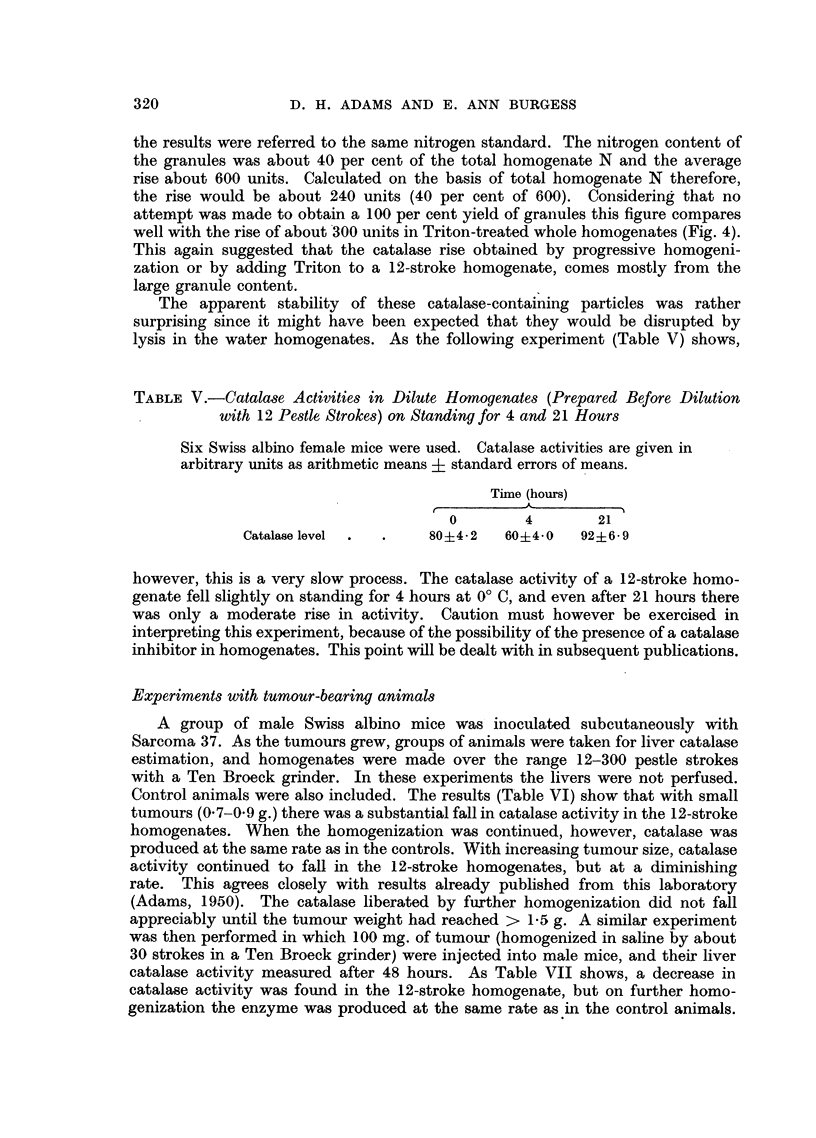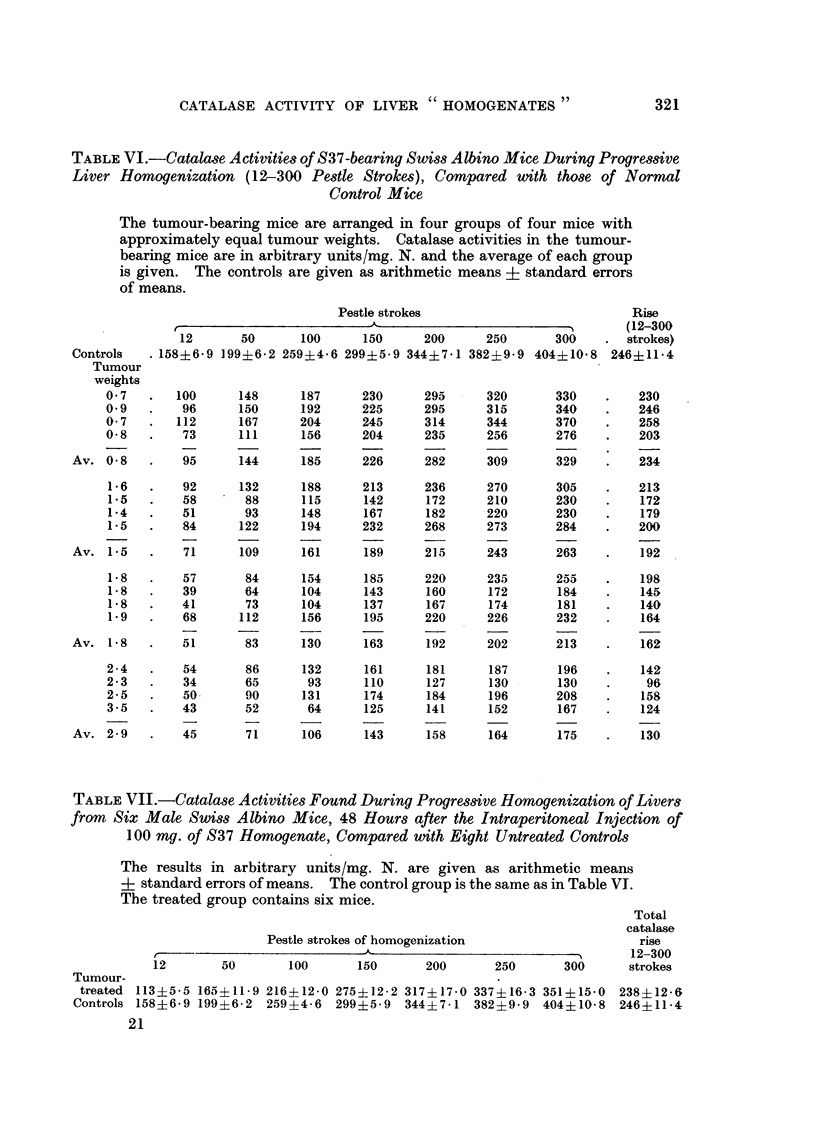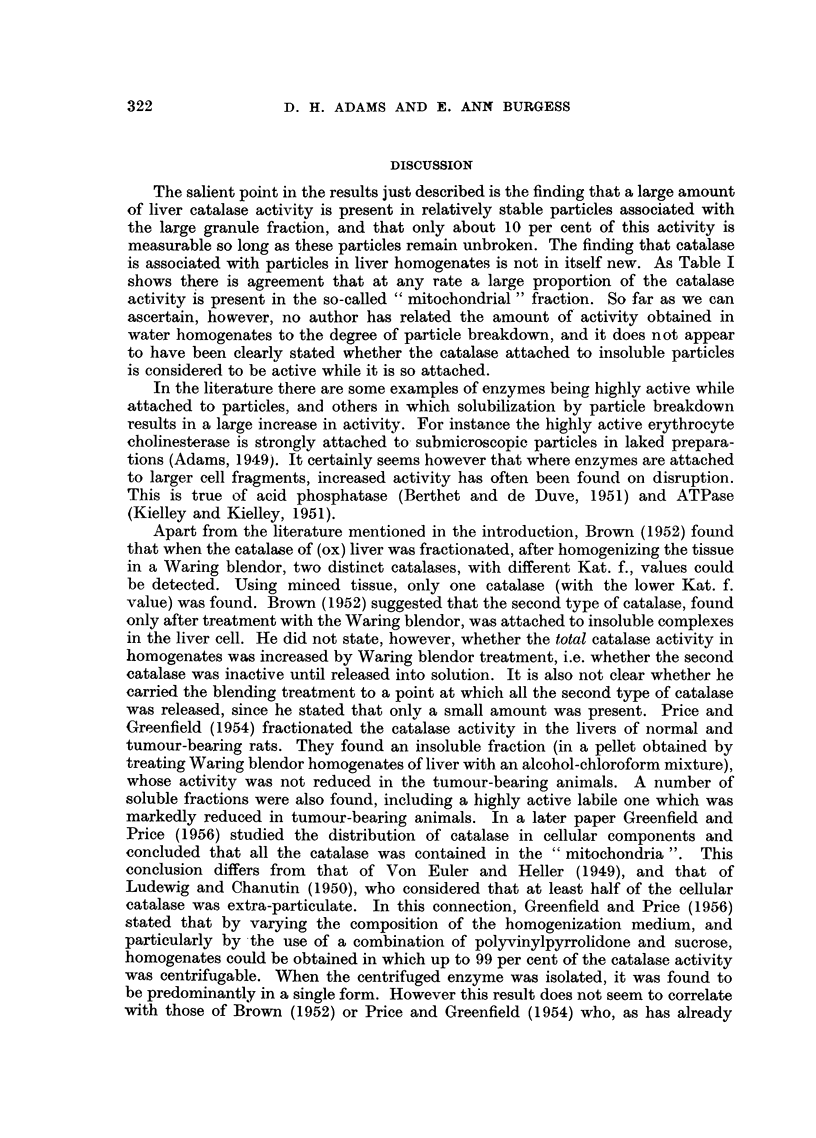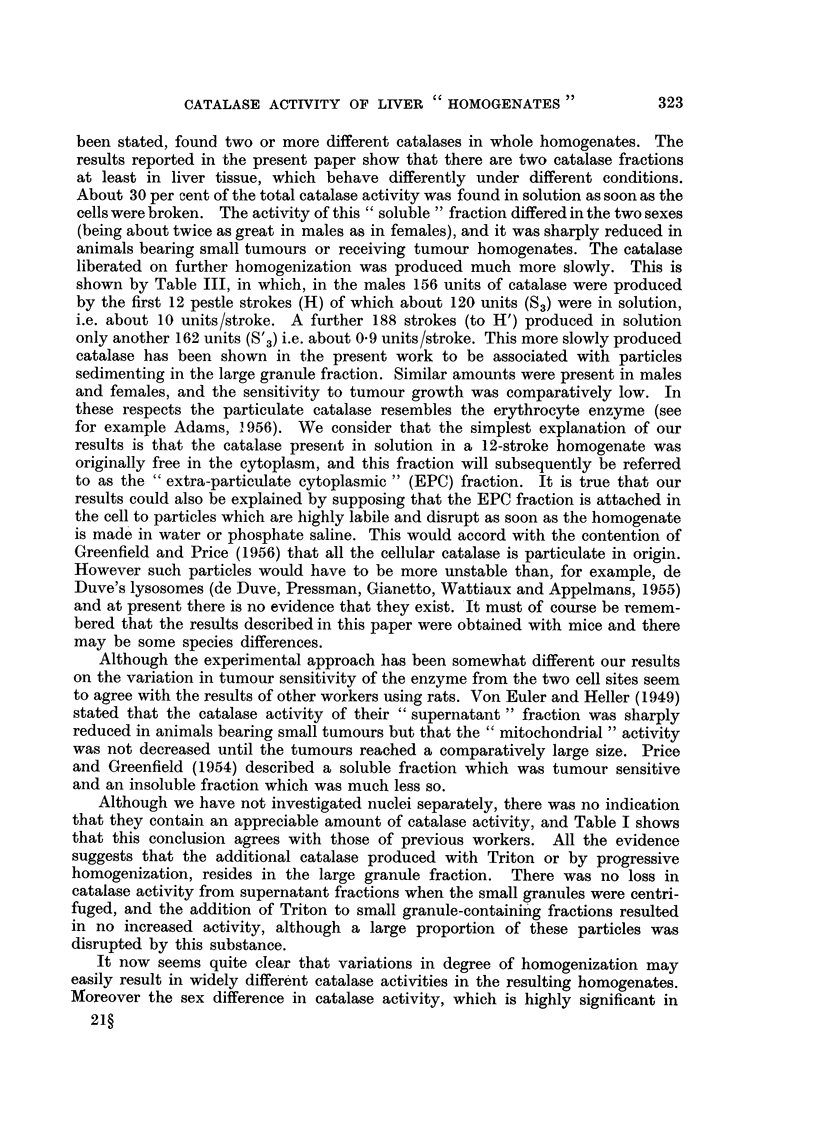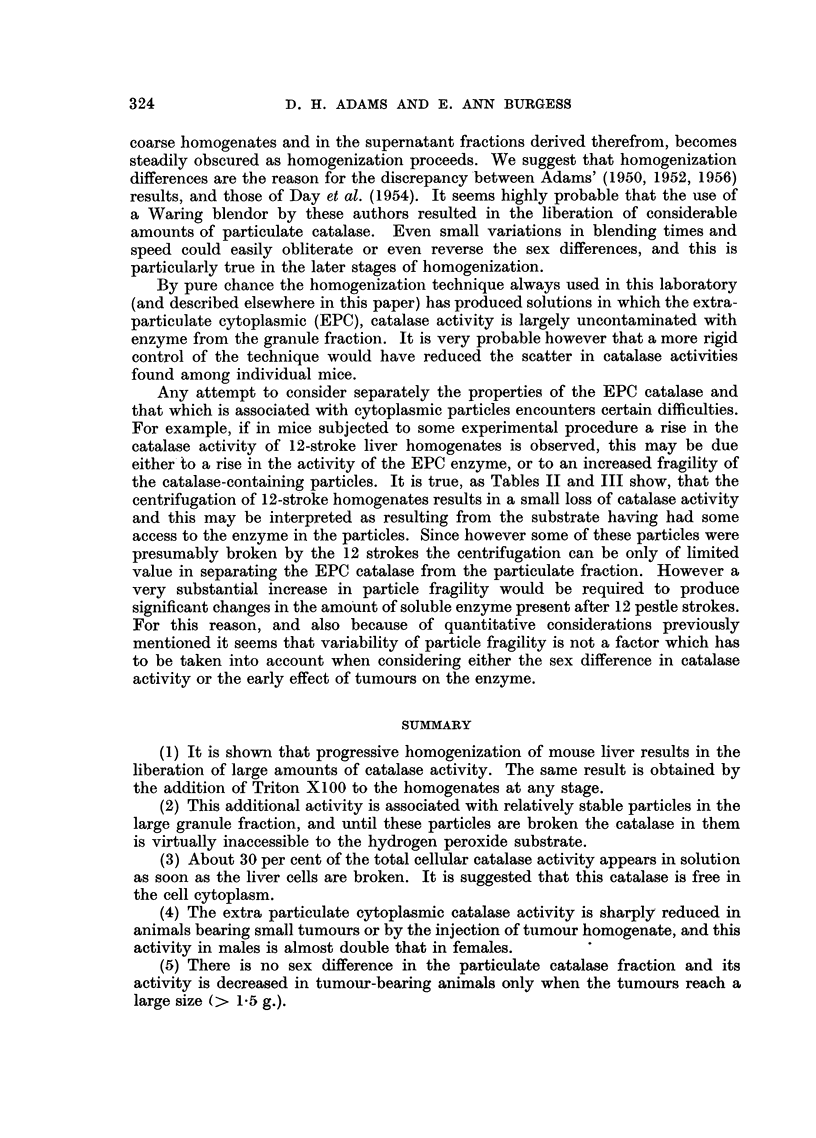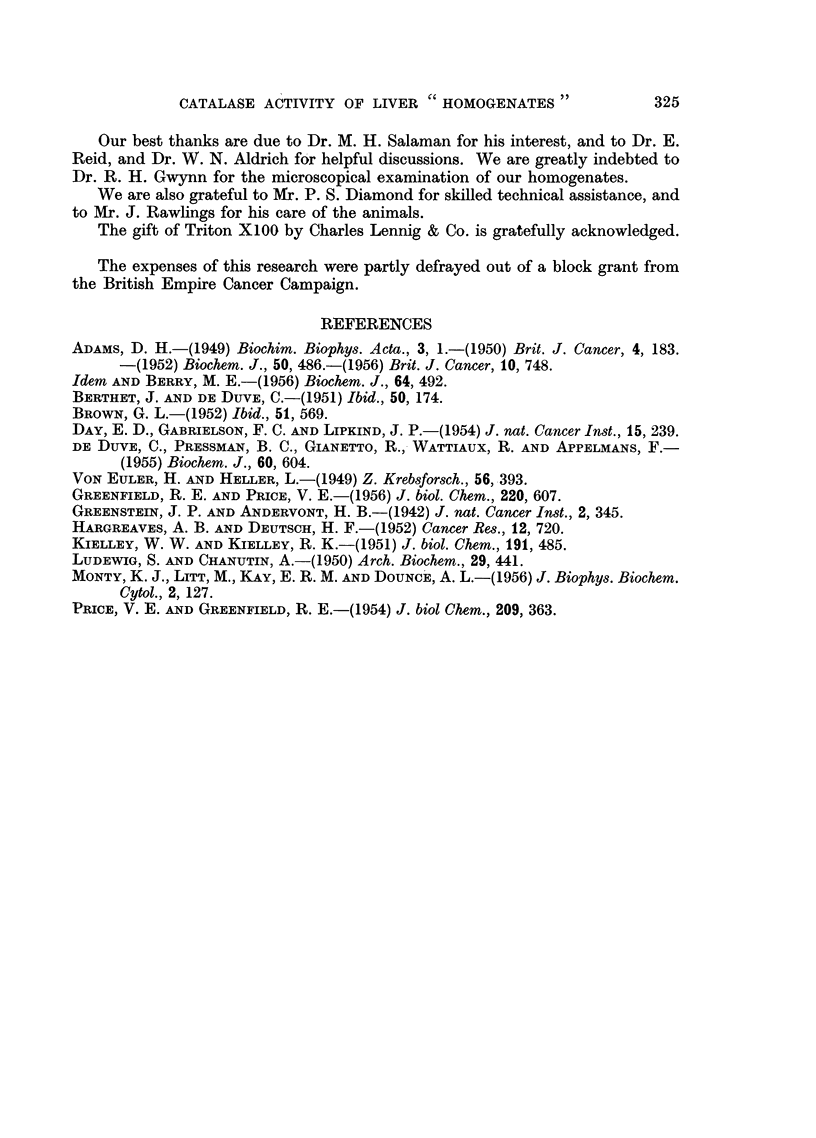# The Effect of the Degree of Homogenization on the Catalase Activity of Liver “Homogenates”

**DOI:** 10.1038/bjc.1957.39

**Published:** 1957-06

**Authors:** D. H. Adams, E. Ann Burgess


### THE EFFECT OF THE DEGREE OF HOMOGENIZATION ON THE CATALASE ACTIVITY OF LIVER " HOMOGENATES "

D. H. ADAMS AND E. ANN BURGESS

*From the Cancer Research Department, London Hospital Medical College, London, E.1*

Received for publication April 18, 1957

THERE seems at present to be no general agreement amongst workers on liver catalase concerning either the best method of estimating the enzyme, or of preparing the homogenates in which the enzyme is measured. It is well known that the results of catalase activity measurements are considerably affected by such variables as temperature, hydrogen peroxide concentration, length of time for which the enzyme and substrate are allowed to remain in contact, and whether the measurements are of oxygen evolution or hydrogen peroxide disappearance. However, little attention seems to have been paid to the possibility that some discrepancies in the results obtained by various authors may depend as much on the method by which liver homogenates are prepared as on the method of enzyme assay.

An example of an apparently complete disagreement in the literature concerns the question of a sex difference in liver catalase activity in mice and rats. Adams (1950, 1952) reported that a sex difference was present in the livers of the heterogenous strains of mice used, the catalase level in males being higher than in females. Hargreaves and Deutsch (1952) found similar results in their rats. However, Day, Gabrielson, and Lipkind (1954) measured liver catalase activities in both sexes of six pure line strains of mice and stated that the male level exceeded the female in only one strain. In three other strains there was no particular sex difference, and in the remaining two the female level was higher than the male. In view of these results Adams (1956) measured catalase activities in the livers of 14 strains of mice (including 10 pure lines) and three pure line strains of rats. All the rats, and all the mice with the exception of the related C57 black and brown strains, had significantly higher liver catalase activities in males than in females. The C57 black strain was stated by Greenstein and Andervont (1942) to be exceptional in that (*a*) the liver catalase activity was insensitive to S37 tumour growth, and (*b*) the activity was only about half that of the other strains studied. Adams (1956) found the catalase level in the C57 strains to be about half that of the other strains, in agreement with Greenstein and Andervont (1942). However, Day *et al.* (1954) stated that the catalase activity of their C57 brown mice was high, and equal to that of the other strains used. They also stated that the catalase levels in all their mice were considerably higher than those obtained by other authors. Greenstein and Andervont prepared their homogenates by grinding livers with sand, and Adams used a Ten Broeck grinder (see methods). Day *et al.*, however, used the more drastic technique of homogenizing in a Waring blendor. It seemed possible therefore that the discrepancies between Greenstein and Andervont's (1942) and Adams' (1950, 1952, 1956) results on the one hand, and those of Day *et al.* (1954) on the other, might be due to the differences in homogenization techniques. Day *et al.* (1954) had themselves suggested that varying homogenization techniques might explain some of the discrepancies between the results obtains by the various workers. *Prime facie* both Greenstein's and Adams' method seemed likely to produce much less tissue disintegration than a Waring blendor. In fact, in the studies previously reported from this laboratory, liver has been homogenized to a point corresponding approximately to the disappearance of intact cells. It was assumed that this procedure would suffice to expose all the catalase in liver to the hydrogen peroxide substrate, particularly since the homogenates were usually made in water. As will be shown, however, this assumption was incorrect, and there is a large amount of catalase activity associated with stable homogenate particles which is not reached by the substrate while the particles remain intact.

It is also relevant to review briefly the literature on the intracellular distribution of liver catalase. As Table I shows, only rats have been used, the sex of the animals has not been mentioned, and there is a certain divergence of opinion concerning the location of the catalase activity and the proportion found at various sites. However there seems to be a qualitative agreement that there is little, if any, catalase in nuclei or " microsomes " and a high proportion of the total in the " mitochondria ".

TABLE I.—*A Brief Summary of the Literature on the Intracellular Distribution of Catalase*

| | | | Catalase activity of | | | |
|---|---|---|---|---|---|---|
| Worker | Animals | Sex | Nuclei | " Mitochondria " | " Microsomes " | Extra particulate cytoplasm |
| Von Euler and Heller, 1949 | Rat | Not stated | Negligible | 20% | *80% | |
| Ludewig and Chanutin, 1950 | " | " | 5% | 45% | — | 49% |
| Monty, Litt, Kay and Dounce, 1956 | " | " | Some | Not investigated | Not investigated | Not investigated |
| Greenfield and Price, 1956 | " | " | — | 100% | — | — |

* These authors did not study the microsome fraction separately.

### MATERIALS AND METHODS

*Animals.*—Young adult mice of the following strains were used :

C3Hb (bred by brother-sister mating in this laboratory from mice originally given to us by the Institute of Cancer Research, Royal Cancer Hospital).

Swiss Albinos (a heterogeneous strain obtained commercially).

The diet of the animals consisted of commercial rat cubes and water (both *ad libitum*).

*Preparation of the liver homogenates.*—Fig. 1 shows a Ten Broeck grinder. The clearance between the pestle and the barrel is approximately 1/40 mm., and the capacity of the barrel about 6 ml. In the standard technique used here the pestle is moved up and down (one stroke) in about 1 second. Consequently at each halfstroke 6 ml. of liquid are forced through the annular space between the pestle and barrel (cross-section 0·9 sq. mm.) in 0·5 sec., giving an average liquid velocity through the space of about 13 metres/sec. The peak liquid velocity must be of the order of double this figure, falling off towards zero at each glass liquid boundary, the velocity of the pestle being negligible in comparison. Compared with rotating pestle homogenizers these velocity gradients are extremely high. Normally one mouse liver is placed in the barrel with about 8 c.c. of water. This excess of fluid

Fig. 1.—A Ten Broeck grinder of the type used in this laboratory. The dimensions are in mm.

reduces the homogenization efficiency since only about two-thirds of the total volume is then swept by the pestle. However, excessive frothing and spurting of liquid occurs unless this is done. Twelve strokes of the pestle give a homogenate containing many free nuclei and granules, but virtually no intact cells. All the catalase work reported previously from this laboratory has been done using homogenates prepared with about 12–16 pestle strokes. In the present work the homogenization has been extended to 300 pestle strokes, and catalase measured at intervals of about 50 strokes.

*Estimation of liver catalase activity.*—Individual livers were homogenized in water and made up to approximately 1 g. liver/10 ml. and then diluted 10 times with water. Where catalase estimations have been made at intervals during homogenization small samples (0·2 ml.) were taken from the grinder for dilution. The catalase activity of the homogenates was then estimated as previously described (Adams, 1950, 1952) and referred to total N. In view of the high activities in some homogenates, 0·1–0·3 ml. of the final dilution has been used, instead of the normal 0·3 ml.

*Tumour*.—Sarcoma 37 (maintained in this laboratory).

*Nuclei counts*.—These were made in an improved Neubauer haemocytometer under phase contrast illumination.

*Triton X*100.—A non-ionic detergent kindly given to us by Charles Lennig & Co. Ltd.

### RESULTS

Fig. 2 shows the effect of progressive homogenization (given as number of strokes of the homogenizer pestle) on the catalase activity of liver from male and female Swiss mice. The homogenization was done at room temperature (20° C.); the reason for this will be given later. The first point on the curves (at 12 pestle strokes) corresponded with the virtual disappearance of intact cells from the homogenate and as Fig. 2 shows, a considerable amount of catalase activity was then present. It will also be seen that a progressive liberation of catalase occurred as far as the homogenization was carried (300 strokes). Although the rate of catalase

FIG. 2.—Catalase activities of livers from male and female Swiss albino mice on progressive homogenization in water and phosphate saline.

Water . . . Males ● ——— ● Females ○——— ○.

Phosphate saline . Males ■——— ■ Females □——— □.

The catalase activities are given, in the water homogenates as arithmetic means ± standard errors of means (six mice per group) and in the phosphate saline homogenates as the arithmetic means of the results obtained from three mice.

*Ordinate* : Catalase activity in arbitrary units/mg. N.
*Abscissa* : Pestle strokes of homogenizer.

production steadily diminished the peak of the curve does not appear to have been reached. It seemed reasonable to suppose that the additional enzyme activity was being liberated from particles broken up by the continued homogenization. Microscopic studies of some of the homogenates were made and showed the following qualitative results. At 12 strokes intact cells were rarely seen but nuclei and granules were abundant. By 150 strokes nuclei were scarce

Fig. 3 (*a*).—Catalase activities of liver from male and female C3Hb mice on progressive homogenization. The closed and open circles respectively represent the catalase levels in individual male and female livers and the crosses show the arithmetic mean values of the groups. Males ●———●. Females ○———○.

(*b*).—Corresponding counts of free nuclei in the homogenates. Each point represents the average of four male and four female mice.

*Ordinate :* (*a*) Catalase activity in arbitrary units/mg. N. (*b*) Free nuclei/$10^{-4}$ mg. N.
*Abscissa :* Pestle strokes of homogenizer.

and were no longer seen at 200 strokes. The large granules steadily disappeared throughout the whole range, and at 300 strokes their concentration had fallen to about one-third of its original value. The comparatively rapid disappearance of nuclei, and the absence of any obvious corresponding break in the curve, suggested that the contribution to the catalase activity produced by rupture of nuclei was probably small. The experiment was repeated with C3Hb mice (Fig. 3), and counts of intact nuclei in the various homogenates were made. The results show clearly that there was no correlation between the disappearance of intact nuclei and the appearance of additional catalase activity. If the homogenization was done at 0° C. the catalase curve was very similar, although perhaps flattened slightly, but the nuclei were much more stable, about 20 per cent of the original number being present after 300 strokes. This gave the misleading appearance, at this temperature, of a positive correlation between loss of nuclei and appearance of catalase.

In a few experiments (three Swiss mice of each sex) the livers were homogenized in the phosphate saline medium used by Adams and Berry (1956) and catalase estimations made at intervals. The averaged results are shown by the dotted lines in Fig. 2 and were closely similar to those obtained by homogenization in water. The slightly lower levels obtained in the saline homogenates may be explained by the persistence of intact cells beyond 12 pestle strokes.

It may also be noted from Fig. 2 and 3 that a sex difference was present in the 12-stroke homogenate, but that the catalase appearing on further homogenization was produced at the same rate in both sexes.

Experiments were then conducted with a view to answering the following questions :

(1) Is the catalase measured in a coarse (12-stroke) homogenate in solution, or is it attached to particles ?

(2) In which fraction does the sex difference in catalase activity occur ?

(3) Assuming that the catalase activity liberated by progressive homogenization comes from the breaking of particles, does the catalase in the unbroken particles make any contribution to the activity of a coarse (12-stroke) homogenate ? This could occur if hydrogen peroxide diffused slowly into the particles, the diffusion rate being the limiting factor.

(4) Is the catalase which appears on further homogenization actually in solution, or is it still attached to small particulate fragments ?

In the normal homogenization technique the livers were not perfused beforehand, and consequently the residual erythrocytes would have contributed to the total catalase activity. While it is generally agreed that this contribution is negligible in ordinary circumstances, in the following experiments the livers were perfused with a phosphate saline before homogenization to remove at any rate the bulk of the erythrocytes.

Livers from male and female C3Hb mice were homogenized (12 pestle strokes), and samples (H) were set aside for catalase and nitrogen estimations. The homogenates were then spun lightly (800 $\times$ g. for 5 minutes) to remove free nuclei and any remaining intact cells. Samples of the supernatant fluid ($S_1$) were also set aside for catalase and nitrogen estimation. These supernatants were then centrifuged (9000 $\times$ g. for 20 minutes) to remove the large granule fraction and after taking samples ($S_2$), were further centrifuged at 24,000 $\times$ g. for 60 minutes ($S_3$). The results appear in Table II. The catalase activities of H and $S_1$ were calculated on their own nitrogen contents, and $S_2$ and $S_3$ on the nitrogen contents of both H and $S_1$ (to indicate how much catalase activity disappeared from the homogenates on centrifuging). In three mice of each sex the catalase activities of the $S_2$ and $S_3$ fractions were calculated on their own nitrogen figures. Table II shows that a sex difference was present in the H homogenates, and that the $S_1$ supernatants (i.e. after spinning down the nuclei and debris), had slightly higher activities than the H homogenates. After spinning down the large granules ($S_2$) there was a catalase loss of 38 units in the males and 30 units in the females (H–$S_2$ with $S_2$ calculated on the H nitrogen), or 29 units in the males and 33 units

TABLE II.—*Catalase Activities in Liver Homogenates after Centrifuging*

The original homogenate (H) was prepared with 12 pestle strokes. $S_1$ is the supernatant after spinning down nuclei and debris and $S_2$ that after spinning down the large granules. $S_3$ is the final supernatant after spinning down the small granules. Six C3Hb mice of each sex were used. Catalase activities are given in arbitrary units/mg. N. as arithmetic means ± std. errors of means.

The H and $S_1$ activities are calculated on their own nitrogen figures and $S_2$ and $S_3$ activities on the nitrogen contents of both H and $S_1$.

| | | | On H nitrogen | | On $S_1$ nitrogen | |
|---|---|---|---|---|---|---|
| | H | $S_1$ | $S_2$ | $S_3$ | $S_2$ | $S_3$ |
| ♂ | 165±5·2 | 177±8·2 | 127±2·9 | 125±3·2 | 148±4·2 | 143±7·1 |
| ♀ | 111±5·3 | 123±7·3 | 81±4·6 | 82±4·7 | 88±5·4 | 87±5·6 |

$S_2$ and $S_3$ activities calculated on their own nitrogen values (three mice of each sex only).

| | $S_2$ | $S_3$ | | $S_2$ | $S_3$ |
|---|---|---|---|---|---|
| ♂ | 211 | 255 | ♀ | 147 | 134 |
| | 246 | 240 | | 124 | 113 |
| | 276 | 267 | | 130 | 156 |
| Av. | 244 | 254 | | 134 | 134 |

in the females ($S_1$–$S_2$ with $S_2$ calculated on the $S_1$ nitrogen). No further loss of activity occurred on spinning down the small granules since the $S_2$ and $S_3$ values are closely similar when calculated on the H or $S_1$ nitrogen contents. Apparently, therefore, the removal of the large granule fraction resulted in the loss from the homogenates of about 30–35 units of catalase activity. This presumably represents the contribution to the total homogenate activity of the unbroken granules. This loss was similar in both sexes, and when taken in conjunction with previous results (e.g. Fig. 2), may be seen to be of the order of 10 per cent of the additional activity produced between 12 and 300 strokes. The male/female activity ratio was higher in the $S_2$ and $S_3$ fractions than in the original (H) homogenates.

Similar results were obtained in a few experiments in which the livers were homogenized in phosphate saline: apparently therefore the bulk of the activity found in 12-stroke homogenates was not centrifugable.

In a further experiment, using Swiss albino mice, the livers from pairs of mice of the same sex were coarsely homogenized (12 strokes) and the homogenates of each pair pooled, and divided into two halves. One half was further homogenized (to 200 strokes) and the centrifuging procedure was repeated with both homogenates. Four pairs of males and four pairs of females were used; the results are given in Table III. The results with the 12-stroke homogenate resembled those obtained with the C3Hb mice (Table II). Considering the 200-stroke homogenates, it is clear that most of the additional activity obtained as a result of the homogenization did not centrifuge down although there was apparently a greater loss of catalase on centrifuging a 200-stroke homogenate than there was from a 12-stroke homogenate. This may be explained by supposing that some particles were damaged sufficiently by the homogenization to increase their permeability to hydrogen peroxide but not sufficiently to liberate their catalase activity into

TABLE III.—*As Table II but with Corresponding Results from 200-stroke Homogenates* (H′ $S'_1$ $S'_2$ $S'_3$)

Each result (12 and 200 strokes) was obtained from a mixed pair of livers (see text). Catalase activities are given in arbitrary units/mg. N. Swiss albino mice were used.

| | 12-stroke | | | | | | | | | 200-stroke | | | | | |
|---|---|---|---|---|---|---|---|---|---|---|---|---|---|---|---|
| | | | On H nitrogen | | On $S_1$ nitrogen | | On own nitrogen | | | | | On H nitrogen | | On $S_1$ nitrogen | |
| | H | $S_1$ | $S_2$ | $S_3$ | $S_2$ | $S_3$ | $S_2$ | $S_3$ | | H′ | $S'_1$ | $S'_2$ | $S'_3$ | $S'_2$ | $S'_3$ |
| | | | | | | | | | ♂ | | | | | | |
| | 158 | 178 | 115 | 112 | 142 | 139 | 210 | 265 | | 380 | 387 | 350 | 350 | 366 | 366 |
| | 151 | 172 | 132 | 126 | 136 | 131 | 204 | 206 | | 343 | 355 | 306 | 296 | 310 | 300 |
| | 159 | 169 | 120 | 118 | 138 | 135 | 202 | 206 | | 301 | 328 | 240 | 218 | 262 | 237 |
| | 156 | 218 | 147 | 138 | 184 | 172 | 230 | 248 | | 325 | 356 | 283 | 276 | 320 | 313 |
| Av. | 156 | 184 | 128 | 123 | 150 | 144 | 212 | 232 | | 337 | 356 | 295 | 285 | 314 | 304 |
| | | | | | | | | | ♀ | | | | | | |
| | 96 | 129 | 88 | 80 | 97 | 88 | 144 | 128 | | 267 | 269 | 198 | 244 | 211 | 240 |
| | 109 | 129 | 80 | 80 | 103 | 103 | 134 | 153 | | 259 | 284 | 239 | 244 | 269 | 233 |
| | 114 | 139 | 89 | 82 | 107 | 98 | 131 | 141 | | 258 | 283 | 205 | 190 | 219 | 204 |
| | 93 | 105 | 70 | 64 | 76 | 68 | 116 | 112 | | 234 | 255 | 171 | 160 | 178 | 168 |
| Av. | 103 | 125 | 82 | 76 | 96 | 89 | 131 | 134 | | 254 | 273 | 203 | 199 | 219 | 211 |

solution. The answers to the questions asked earlier appeared therefore to be as follows : (1) and (3) The catalase activity in a coarse homogenate is largely extra particulate but the unbroken particles in coarse homogenates contribute about 30–35 arbitrary units/mg. N to the total. (2) The catalase sex difference is present in the extra particulate fraction in coarse homogenates. (4) The catalase liberated on further homogenization is largely in solution.

From the curves in Fig. 2 and 3 it appeared that the catalase level had not reached its highest value after 300 pestle strokes of homogenization. Experiments were then done to see whether the enzyme could be liberated in other ways. At the suggestion of Dr. E. Reid we tried the effect on catalase liberation of adding Triton X100 to liver homogenates. Fig. 4 shows the effect of a final concentration of 1 per cent Triton on the catalase activity of liver homogenates from males at various stages, and for comparison, from female liver homogenates prepared with 12 pestle strokes only. Swiss albino mice were used. The Triton was added to the dilute homogenates (see "Methods") immediately before the catalase was estimated. Sighting experiments suggested that a Triton concentration range of 0·5–2 per cent had little effect on the amount of catalase liberated, and no increased activity was observed when the treated homogenates were allowed to stand for up to 5 minutes. The results in Fig. 4 show that at all stages of homogenization from 12–300 strokes the addition of Triton resulted in an elevation of catalase activity to a level a little above that resulting from 300 strokes of homogenization. This suggested that the Triton was acting in a way essentially similar to prolonged homogenization, and also that the level reached by 300 pestle strokes was not far short of the maximum. The results also show that the difference in activity between the 12-stroke male and female homogenates was 65 units both before and after addition of Triton. That Triton itself had a negligible inhibitory effect

FIG. 4.—The effect on catalase activity of the addition of 1 per cent Triton X100 to liver homogenates at various stages of homogenization (males) and to a 12-stroke homogenate (females).

● ——— ● Males homogenized (no Triton).

▲ ——— ▲ Triton added to male homogenates at various stages.

○ Females, 12-stroke homogenate (no Triton).

△ Females, 12-stroke homogenate (after Triton). Catalase activities are given as arithmetic means $\pm$ standard errors of means. Six mice per group.

*Ordinate* : Catalase activity in arbitrary units/mg.N.

*Abscissa* : Pestle strokes of homogenizer.

on catalase is shown in Table IV where up to 2 per cent was added to the supernatant from a centrifuged (9000 $\times$ g. for 20 minutes) homogenate. This result also indicated that the Triton produced no liberation of catalase in the absence of the large granule cell fraction. It is possible that in this experiment a small amount of the catalase liberated was masking a slight enzyme inhibition, but this is unlikely to be more than a small effect, if indeed it occurs at all.

An interesting difference between a 12-stroke homogenate treated with Triton and a 300-stroke homogenate was the persistence of intact nuclei in the former, examined microscopically. This again suggested (cf. Fig. 3) that the breakdown of cell nuclei played little part in the process of catalase liberation. In view of this, and owing to the extreme difficulty of obtaining cell nuclei free from contamination with large granules, it was decided to prepare samples of the large granule fraction. This experiment was done so that the effect of Triton could be studied on the large granule and supernatant fractions separately, and to confirm that the granule fraction is the source of the additional catalase activity.

Coarse (12-stroke) liver homogenates were centrifuged (800 × g. for 5 minutes) to remove nuclei and large fragments ; the supernatant was removed and again spun (9000 × g. for 20 minutes) and the new supernatant removed from the large granule fraction. Under these conditions the small granule fraction was still present in the supernatant. It was decided not to attempt to wash the large granules, in case this should result in variable losses of catalase through particle

FIG. 5.—The effect of the addition of up to 2 per cent Triton X100 on the catalase activity of a centrifuged homogenate. The dots represent the average of three experiments on the same preparation. No inhibitory effect was observed.
*Ordinate :* Catalase activity in arbitrary units.
*Abscissa :* Final per cent Triton.

disruption. The large granules were then re-suspended in water (two pestle strokes with a Ten Broeck grinder) and the volume of the suspensions made approximately equal to the volume of original liver homogenate. The final supernatant and re-suspended granule fractions from each liver were then each divided into two parts, and after the usual 1 : 10 dilution, Triton was added to one part only, to a final concentration of 1 per cent. Catalase estimations were then made on all the samples, and the results are given in Table IV. In both the large granule and supernatant fractions, catalase activities were calculated on their own nitrogen contents. It will be seen that the sex difference was present in the supernatants, and that the addition to them of Triton caused no catalase liberation. Microscopical examination of these supernatant fractions showed that a large proportion of the small granules disappeared after the Triton treatment.

TABLE IV.—*Liver Catalase Activities in Large Granule and Supernatant Fractions, Before and After the Addition of Triton X100*

The supernatant fractions contained the small granules. Six Swiss albino mice of each sex were used. Catalase activities are given in arbitrary units/mg. N., as arithmetic means ± standard errors of means.

| | ♂ | | ♀ | |
|---|---|---|---|---|
| | Large granules | Supernatant | Large granules | Supernatant |
| No Triton | 84±4·5 | 202±12·0 | 77±3·0 | 114±7·4 |
| 1% Triton | 663±36 | 198±11·5 | 700±28 | 123±8·4 |

In the large granule fraction however, no significant sex difference was present before or after the addition of Triton, and the Triton liberated a large amount of catalase. As in the supernatant fractions, many granules disappeared when the Triton was added.

The rise in catalase activity in the large granules (after the addition of Triton) was approximately that which would be expected in a whole homogenate, when the results were referred to the same nitrogen standard. The nitrogen content of the granules was about 40 per cent of the total homogenate N and the average rise about 600 units. Calculated on the basis of total homogenate N therefore, the rise would be about 240 units (40 per cent of 600). Considering that no attempt was made to obtain a 100 per cent yield of granules this figure compares well with the rise of about 300 units in Triton-treated whole homogenates (Fig. 4). This again suggested that the catalase rise obtained by progressive homogenization or by adding Triton to a 12-stroke homogenate, comes mostly from the large granule content.

The apparent stability of these catalase-containing particles was rather surprising since it might have been expected that they would be disrupted by lysis in the water homogenates. As the following experiment (Table V) shows,

TABLE V.—*Catalase Activities in Dilute Homogenates (Prepared Before Dilution with 12 Pestle Strokes) on Standing for 4 and 21 Hours*

Six Swiss albino female mice were used. Catalase activities are given in arbitrary units as arithmetic means ± standard errors of means.

| | Time (hours) | | |
|---|---|---|---|
| | 0 | 4 | 21 |
| Catalase level . . | 80±4·2 | 60±4·0 | 92±6·9 |

however, this is a very slow process. The catalase activity of a 12-stroke homogenate fell slightly on standing for 4 hours at 0° C, and even after 21 hours there was only a moderate rise in activity. Caution must however be exercised in interpreting this experiment, because of the possibility of the presence of a catalase inhibitor in homogenates. This point will be dealt with in subsequent publications.

*Experiments with tumour-bearing animals*

A group of male Swiss albino mice was inoculated subcutaneously with Sarcoma 37. As the tumours grew, groups of animals were taken for liver catalase estimation, and homogenates were made over the range 12–300 pestle strokes with a Ten Broeck grinder. In these experiments the livers were not perfused. Control animals were also included. The results (Table VI) show that with small tumours (0·7–0·9 g.) there was a substantial fall in catalase activity in the 12-stroke homogenates. When the homogenization was continued, however, catalase was produced at the same rate as in the controls. With increasing tumour size, catalase activity continued to fall in the 12-stroke homogenates, but at a diminishing rate. This agrees closely with results already published from this laboratory (Adams, 1950). The catalase liberated by further homogenization did not fall appreciably until the tumour weight had reached > 1·5 g. A similar experiment was then performed in which 100 mg. of tumour (homogenized in saline by about 30 strokes in a Ten Broeck grinder) were injected into male mice, and their liver catalase activity measured after 48 hours. As Table VII shows, a decrease in catalase activity was found in the 12-stroke homogenate, but on further homogenization the enzyme was produced at the same rate as in the control animals.

TABLE VI.—*Catalase Activities of S37-bearing Swiss Albino Mice During Progressive Liver Homogenization (12–300 Pestle Strokes), Compared with those of Normal Control Mice*

The tumour-bearing mice are arranged in four groups of four mice with approximately equal tumour weights. Catalase activities in the tumour-bearing mice are in arbitrary units/mg. N. and the average of each group is given. The controls are given as arithmetic means ± standard errors of means.

| | Pestle strokes | | | | | | | Rise |
|---|---|---|---|---|---|---|---|---|
| | 12 | 50 | 100 | 150 | 200 | 250 | 300 | (12–300 strokes) |
| Controls | 158±6·9 | 199±6·2 | 259±4·6 | 299±5·9 | 344±7·1 | 382±9·9 | 404±10·8 | 246±11·4 |
| Tumour weights | | | | | | | | |
| 0·7 | 100 | 148 | 187 | 230 | 295 | 320 | 330 | 230 |
| 0·9 | 96 | 150 | 192 | 225 | 295 | 315 | 340 | 246 |
| 0·7 | 112 | 167 | 204 | 245 | 314 | 344 | 370 | 258 |
| 0·8 | 73 | 111 | 156 | 204 | 235 | 256 | 276 | 203 |
| Av. 0·8 | 95 | 144 | 185 | 226 | 282 | 309 | 329 | 234 |
| 1·6 | 92 | 132 | 188 | 213 | 236 | 270 | 305 | 213 |
| 1·5 | 58 | 88 | 115 | 142 | 172 | 210 | 230 | 172 |
| 1·4 | 51 | 93 | 148 | 167 | 182 | 220 | 230 | 179 |
| 1·5 | 84 | 122 | 194 | 232 | 268 | 273 | 284 | 200 |
| Av. 1·5 | 71 | 109 | 161 | 189 | 215 | 243 | 263 | 192 |
| 1·8 | 57 | 84 | 154 | 185 | 220 | 235 | 255 | 198 |
| 1·8 | 39 | 64 | 104 | 143 | 160 | 172 | 184 | 145 |
| 1·8 | 41 | 73 | 104 | 137 | 167 | 174 | 181 | 140 |
| 1·9 | 68 | 112 | 156 | 195 | 220 | 226 | 232 | 164 |
| Av. 1·8 | 51 | 83 | 130 | 163 | 192 | 202 | 213 | 162 |
| 2·4 | 54 | 86 | 132 | 161 | 181 | 187 | 196 | 142 |
| 2·3 | 34 | 65 | 93 | 110 | 127 | 130 | 130 | 96 |
| 2·5 | 50 | 90 | 131 | 174 | 184 | 196 | 208 | 158 |
| 3·5 | 43 | 52 | 64 | 125 | 141 | 152 | 167 | 124 |
| Av. 2·9 | 45 | 71 | 106 | 143 | 158 | 164 | 175 | 130 |

TABLE VII.—*Catalase Activities Found During Progressive Homogenization of Livers from Six Male Swiss Albino Mice, 48 Hours after the Intraperitoneal Injection of 100 mg. of S37 Homogenate, Compared with Eight Untreated Controls*

The results in arbitrary units/mg. N. are given as arithmetic means ± standard errors of means. The control group is the same as in Table VI. The treated group contains six mice.

| | Pestle strokes of homogenization | | | | | | | Total catalase rise 12–300 strokes |
|---|---|---|---|---|---|---|---|---|
| | 12 | 50 | 100 | 150 | 200 | 250 | 300 | |
| Tumour-treated | 113±5·5 | 165±11·9 | 216±12·0 | 275±12·2 | 317±17·0 | 337±16·3 | 351±15·0 | 238±12·6 |
| Controls | 158±6·9 | 199±6·2 | 259±4·6 | 299±5·9 | 344±7·1 | 382±9·9 | 404±10·8 | 246±11·4 |

### DISCUSSION

The salient point in the results just described is the finding that a large amount of liver catalase activity is present in relatively stable particles associated with the large granule fraction, and that only about 10 per cent of this activity is measurable so long as these particles remain unbroken. The finding that catalase is associated with particles in liver homogenates is not in itself new. As Table I shows there is agreement that at any rate a large proportion of the catalase activity is present in the so-called " mitochondrial " fraction. So far as we can ascertain, however, no author has related the amount of activity obtained in water homogenates to the degree of particle breakdown, and it does not appear to have been clearly stated whether the catalase attached to insoluble particles is considered to be active while it is so attached.

In the literature there are some examples of enzymes being highly active while attached to particles, and others in which solubilization by particle breakdown results in a large increase in activity. For instance the highly active erythrocyte cholinesterase is strongly attached to submicroscopic particles in laked preparations (Adams, 1949). It certainly seems however that where enzymes are attached to larger cell fragments, increased activity has often been found on disruption. This is true of acid phosphatase (Berthet and de Duve, 1951) and ATPase (Kielley and Kielley, 1951).

Apart from the literature mentioned in the introduction, Brown (1952) found that when the catalase of (ox) liver was fractionated, after homogenizing the tissue in a Waring blendor, two distinct catalases, with different Kat. f., values could be detected. Using minced tissue, only one catalase (with the lower Kat. f. value) was found. Brown (1952) suggested that the second type of catalase, found only after treatment with the Waring blendor, was attached to insoluble complexes in the liver cell. He did not state, however, whether the *total* catalase activity in homogenates was increased by Waring blendor treatment, i.e. whether the second catalase was inactive until released into solution. It is also not clear whether he carried the blending treatment to a point at which all the second type of catalase was released, since he stated that only a small amount was present. Price and Greenfield (1954) fractionated the catalase activity in the livers of normal and tumour-bearing rats. They found an insoluble fraction (in a pellet obtained by treating Waring blendor homogenates of liver with an alcohol-chloroform mixture), whose activity was not reduced in the tumour-bearing animals. A number of soluble fractions were also found, including a highly active labile one which was markedly reduced in tumour-bearing animals. In a later paper Greenfield and Price (1956) studied the distribution of catalase in cellular components and concluded that all the catalase was contained in the " mitochondria ". This conclusion differs from that of Von Euler and Heller (1949), and that of Ludewig and Chanutin (1950), who considered that at least half of the cellular catalase was extra-particulate. In this connection, Greenfield and Price (1956) stated that by varying the composition of the homogenization medium, and particularly by the use of a combination of polyvinylpyrrolidone and sucrose, homogenates could be obtained in which up to 99 per cent of the catalase activity was centrifugable. When the centrifuged enzyme was isolated, it was found to be predominantly in a single form. However this result does not seem to correlate with those of Brown (1952) or Price and Greenfield (1954) who, as has already been stated, found two or more different catalases in whole homogenates. The results reported in the present paper show that there are two catalase fractions at least in liver tissue, which behave differently under different conditions. About 30 per cent of the total catalase activity was found in solution as soon as the cells were broken. The activity of this " soluble " fraction differed in the two sexes (being about twice as great in males as in females), and it was sharply reduced in animals bearing small tumours or receiving tumour homogenates. The catalase liberated on further homogenization was produced much more slowly. This is shown by Table III, in which, in the males 156 units of catalase were produced by the first 12 pestle strokes (H) of which about 120 units ($S_3$) were in solution, i.e. about 10 units/stroke. A further 188 strokes (to H′) produced in solution only another 162 units ($S'_3$) i.e. about 0·9 units/stroke. This more slowly produced catalase has been shown in the present work to be associated with particles sedimenting in the large granule fraction. Similar amounts were present in males and females, and the sensitivity to tumour growth was comparatively low. In these respects the particulate catalase resembles the erythrocyte enzyme (see for example Adams, 1956). We consider that the simplest explanation of our results is that the catalase present in solution in a 12-stroke homogenate was originally free in the cytoplasm, and this fraction will subsequently be referred to as the " extra-particulate cytoplasmic " (EPC) fraction. It is true that our results could also be explained by supposing that the EPC fraction is attached in the cell to particles which are highly labile and disrupt as soon as the homogenate is made in water or phosphate saline. This would accord with the contention of Greenfield and Price (1956) that all the cellular catalase is particulate in origin. However such particles would have to be more unstable than, for example, de Duve's lysosomes (de Duve, Pressman, Gianetto, Wattiaux and Appelmans, 1955) and at present there is no evidence that they exist. It must of course be remembered that the results described in this paper were obtained with mice and there may be some species differences.

Although the experimental approach has been somewhat different our results on the variation in tumour sensitivity of the enzyme from the two cell sites seem to agree with the results of other workers using rats. Von Euler and Heller (1949) stated that the catalase activity of their " supernatant " fraction was sharply reduced in animals bearing small tumours but that the " mitochondrial " activity was not decreased until the tumours reached a comparatively large size. Price and Greenfield (1954) described a soluble fraction which was tumour sensitive and an insoluble fraction which was much less so.

Although we have not investigated nuclei separately, there was no indication that they contain an appreciable amount of catalase activity, and Table I shows that this conclusion agrees with those of previous workers. All the evidence suggests that the additional catalase produced with Triton or by progressive homogenization, resides in the large granule fraction. There was no loss in catalase activity from supernatant fractions when the small granules were centrifuged, and the addition of Triton to small granule-containing fractions resulted in no increased activity, although a large proportion of these particles was disrupted by this substance.

It now seems quite clear that variations in degree of homogenization may easily result in widely different catalase activities in the resulting homogenates. Moreover the sex difference in catalase activity, which is highly significant in coarse homogenates and in the supernatant fractions derived therefrom, becomes steadily obscured as homogenization proceeds. We suggest that homogenization differences are the reason for the discrepancy between Adams' (1950, 1952, 1956) results, and those of Day *et al.* (1954). It seems highly probable that the use of a Waring blendor by these authors resulted in the liberation of considerable amounts of particulate catalase. Even small variations in blending times and speed could easily obliterate or even reverse the sex differences, and this is particularly true in the later stages of homogenization.

By pure chance the homogenization technique always used in this laboratory (and described elsewhere in this paper) has produced solutions in which the extraparticulate cytoplasmic (EPC), catalase activity is largely uncontaminated with enzyme from the granule fraction. It is very probable however that a more rigid control of the technique would have reduced the scatter in catalase activities found among individual mice.

Any attempt to consider separately the properties of the EPC catalase and that which is associated with cytoplasmic particles encounters certain difficulties. For example, if in mice subjected to some experimental procedure a rise in the catalase activity of 12-stroke liver homogenates is observed, this may be due either to a rise in the activity of the EPC enzyme, or to an increased fragility of the catalase-containing particles. It is true, as Tables II and III show, that the centrifugation of 12-stroke homogenates results in a small loss of catalase activity and this may be interpreted as resulting from the substrate having had some access to the enzyme in the particles. Since however some of these particles were presumably broken by the 12 strokes the centrifugation can be only of limited value in separating the EPC catalase from the particulate fraction. However a very substantial increase in particle fragility would be required to produce significant changes in the amount of soluble enzyme present after 12 pestle strokes. For this reason, and also because of quantitative considerations previously mentioned it seems that variability of particle fragility is not a factor which has to be taken into account when considering either the sex difference in catalase activity or the early effect of tumours on the enzyme.

### SUMMARY

(1) It is shown that progressive homogenization of mouse liver results in the liberation of large amounts of catalase activity. The same result is obtained by the addition of Triton X100 to the homogenates at any stage.

(2) This additional activity is associated with relatively stable particles in the large granule fraction, and until these particles are broken the catalase in them is virtually inaccessible to the hydrogen peroxide substrate.

(3) About 30 per cent of the total cellular catalase activity appears in solution as soon as the liver cells are broken. It is suggested that this catalase is free in the cell cytoplasm.

(4) The extra particulate cytoplasmic catalase activity is sharply reduced in animals bearing small tumours or by the injection of tumour homogenate, and this activity in males is almost double that in females.

(5) There is no sex difference in the particulate catalase fraction and its activity is decreased in tumour-bearing animals only when the tumours reach a large size ($>$ 1·5 g.).

Our best thanks are due to Dr. M. H. Salaman for his interest, and to Dr. E. Reid, and Dr. W. N. Aldrich for helpful discussions. We are greatly indebted to Dr. R. H. Gwynn for the microscopical examination of our homogenates.

We are also grateful to Mr. P. S. Diamond for skilled technical assistance, and to Mr. J. Rawlings for his care of the animals.

The gift of Triton X100 by Charles Lennig & Co. is gratefully acknowledged.

The expenses of this research were partly defrayed out of a block grant from the British Empire Cancer Campaign.

### REFERENCES

Adams, D. H.—(1949) *Biochim. Biophys. Acta.*, **3**, 1.—(1950) *Brit. J. Cancer*, **4**, 183. —(1952) *Biochem. J.*, **50**, 486.—(1956) *Brit. J. Cancer*, **10**, 748.

*Idem* and Berry, M. E.—(1956) *Biochem. J.*, **64**, 492.

Berthet, J. and de Duve, C.—(1951) *Ibid.*, **50**, 174.

Brown, G. L.—(1952) *Ibid.*, **51**, 569.

Day, E. D., Gabrielson, F. C. and Lipkind, J. P.—(1954) *J. nat. Cancer Inst.*, **15**, 239.

de Duve, C., Pressman, B. C., Gianetto, R., Wattiaux, R. and Appelmans, F.—(1955) *Biochem. J.*, **60**, 604.

Von Euler, H. and Heller, L.—(1949) *Z. Krebsforsch.*, **56**, 393.

Greenfield, R. E. and Price, V. E.—(1956) *J. biol. Chem.*, **220**, 607.

Greenstein, J. P. and Andervont, H. B.—(1942) *J. nat. Cancer Inst.*, **2**, 345.

Hargreaves, A. B. and Deutsch, H. F.—(1952) *Cancer Res.*, **12**, 720.

Kielley, W. W. and Kielley, R. K.—(1951) *J. biol. Chem.*, **191**, 485.

Ludewig, S. and Chanutin, A.—(1950) *Arch. Biochem.*, **29**, 441.

Monty, K. J., Litt, M., Kay, E. R. M. and Dounce, A. L.—(1956) *J. Biophys. Biochem. Cytol.*, **2**, 127.

Price, V. E. and Greenfield, R. E.—(1954) *J. biol Chem.*, **209**, 363.